# Transcranial cortex-wide Ca^2+^ imaging for the functional mapping of cortical dynamics

**DOI:** 10.3389/fnins.2023.1119793

**Published:** 2023-02-15

**Authors:** Serika Yamada, Yan Wang, Hiromu Monai

**Affiliations:** ^1^Department of Biology, Faculty of Science, Ochanomizu University, Tokyo, Japan; ^2^Graduate School of Humanities and Sciences, Ochanomizu University, Tokyo, Japan

**Keywords:** neuron, astrocytes, Ca^2+^ imaging, wide-field, genetically encoded calcium indicators (GECI)

## Abstract

Visualization and tracking of the information flow in the broader brain area are essential because nerve cells make a vast network in the brain. Fluorescence Ca^2+^ imaging is a simultaneous visualization of brain cell activities in a wide area. Instead of classical chemical indicators, developing various types of transgenic animals that express Ca^2+^-sensitive fluorescent proteins enables us to observe brain activities in living animals at a larger scale for a long time. Multiple kinds of literature have reported that transcranial imaging of such transgenic animals is practical for monitoring the wide-field information flow across the broad brain regions, although it has a lower spatial resolution. Notably, this technique is helpful for the initial evaluation of cortical function in disease models. This review will introduce fully intact transcranial macroscopic imaging and cortex-wide Ca^2+^ imaging as practical applications.

## 1. Introduction

The brain is often considered a black box, as its internal functions cannot be entirely deduced from external observations alone. Nevertheless, the brain’s electromagnetic activities can be visualized and quantified using electroencephalography (EEG) and magnetoencephalography (MEG). Additionally, changes in blood flow and metabolic activity can be detected through functional magnetic resonance imaging (fMRI) and positron emission tomography (PET) techniques.

Neurons, a type of brain cell, mediate electrical information through membrane potentials, the activity of which can be quantified *via* electrophysiological measurements using electrodes. While these measurements offer a high degree of temporal precision, their spatial resolution is often limited. Implementing monitoring techniques with an enhanced spatial resolution is essential to fully comprehend the causal interactions between individual neurons within neural networks.

Fluorescence imaging is a method for simultaneously visualizing neural activities, such as changes in membrane potentials and intracellular calcium ion (Ca^2+^) concentrations, within a field of view through fluorescence-tagged sensors. A key advantage of this technique is non-invasive, as it utilizes photons rather than penetrating electrodes. Importantly, it is well-established that neurons increase their intracellular Ca^2+^ concentrations when its membrane depolarized.

Furthermore, assessing the activity of astrocytes, a type of non-neuronal brain cell, through electrophysiological means is particularly challenging as their membrane potential changes are less active than neurons. As a result, the contribution of astrocytes to dynamic brain functions such as information processing has long been underappreciated and considered just supporting cells. Nevertheless, recent studies using calcium fluorescence imaging revealed that astrocytes also exhibit dynamic intracellular Ca^2+^ elevation, suggesting a more active role in neural processing (Araque et al., 2001).

Using a chemical indicator for *in vivo* Ca^2+^ imaging was historically invasive, as it required the implementation of a loading electrode to inject the dye into brain tissue (Stosiek et al., 2003). Furthermore, it should be noted that some Ca^2+^ indicators, such as Fluo-4, Rhod-2, and Fura-2, can cause significant cellular toxicity (Smith et al., 2018). While moderate concentrations may not be toxic when used appropriately, it is important to exercise caution when handling these compounds and to conduct additional control experiments (Martin and Bernard, 2018). In contrast, advancements in transgenic animal research have yielded the development of various strains expressing Ca^2+^-sensitive fluorescent proteins, allowing for non-invasive, less toxic, and sustained observation of brain activity in living subjects (Smith et al., 2018).

The highly scattering nature of brain tissue hinders the penetration of visible wavelengths to deeper brain regions. For example, the thinnest portion of the cerebral cortex in mice is roughly 800–900 μm; however, traditional epifluorescence microscopy can only visualize up to a few hundred micrometers beneath the surface of the brain using single photon illumination. In contrast, two-photon microscopy, which uses a specialized scanning laser, enables observation of deeper regions exceeding 800 μm from the surface (Kawakami et al., 2013; Ue et al., 2018). The limited focal plane in two-photon microscopy is particularly advantageous for analyzing neural network structure and the spatiotemporal relationships between neurons and astrocytes.

Visualizing and tracking information flow across multiple and extensive fields is crucial as nerve cells constitute a vast network within the brain. Functional imaging techniques, such as fMRI and PET, commonly utilized non-invasive macroscopic imaging tools, allow for the visualization of metabolic changes following neural activity through contrast agents to enhance signals. However, these techniques necessitate specialized and costly equipment. In contrast, recent advancements in two-photon microscopy have enabled imaging with a field of view that is substantially larger, spanning a few millimeters, sufficient for the simultaneous visualization of over 10,000 cells (Sofroniew et al., 2016; Stringer et al., 2019a,b). However, conventional two-photon microscopy has traditionally required invasive techniques such as the open-skull method or craniotomy to monitor cellular activity, which can damage brain tissue.

The open-skull method is a highly technical surgical procedure involving removing a portion of the skull (typically less than 0.2 mm) and, in some cases, the dura and the subsequent implantation of a coverslip. Mastery of this technique requires a significant investment of time and training. Additionally, using an electric drill to remove the skull can cause tissue damage and severe bleeding. An alternative to the open-skull method is the thinning of the skull, although it remains technically challenging.

Recent advancements in transgenic technologies have enabled the monitoring of brain activity through the skull through transcranial imaging. While possessing a relatively lower spatial resolution, it boasts a larger field of view to the extent possible for functional imaging. In addition, various studies have reported the practicality of transcranial imaging for monitoring wide-field information flow across broad brain regions (Vanni and Murphy, 2014; Silasi et al., 2016; Xie et al., 2016; Vanni et al., 2017; Zhu et al., 2018; O’Hashi et al., 2019; Barson et al., 2020). This review aims to overview the practical applications of fully intact transcranial macroscopic imaging, specifically in cortex-wide Ca^2+^ imaging.

## 2. A brief history of the cortex-wide transcranial Ca^2+^ imaging

Traditionally, transcranial imaging in living animals has been used to visualize autofluorescence changes corresponding to local metabolic changes through intrinsic optical imaging (Schuett et al., 2002; Kalatsky and Stryker, 2003; Zepeda et al., 2004) and flavin-based fluorescent protein imaging (Takahashi et al., 2006; Tohmi et al., 2009). In addition, the use of large craniotomies enabled hemisphere-wide imaging through the use of membrane voltage-sensitive dyes (Ferezou et al., 2007; Mohajerani et al., 2013) and Ca^2+^ sensors (Stroh et al., 2013; Matsui et al., 2016). In the case of non-invasive imaging, the thinning of the skull in transgenic mice has also enabled cortex-wide imaging (Carandini et al., 2015; Ma et al., 2016; Chen et al., 2017; Rossi et al., 2017).

Vanni and Murphy (2014) reported the first instance of fully intact transcranial imaging with transgenic mice that express a genetically encoded calcium indicator (GECI), specifically GCaMP3. They employed dental cement to seal the surface of the cranial bone to preserve transparency and used a large cover glass to enable chronic monitoring (Vanni and Murphy, 2014; Silasi et al., 2016). Subsequently, transcranial imaging was established to observe cortex-wide functional connectivities across a broad range of brain regions (Vanni and Murphy, 2014; Silasi et al., 2016; Xie et al., 2016; Vanni et al., 2017; Zhu et al., 2018; O’Hashi et al., 2019; Barson et al., 2020), especially for multiple sensory modalities, such as visual (Murphy et al., 2016; Clancy et al., 2019; Pinto et al., 2019; Shimaoka et al., 2019; Salkoff et al., 2020; Sit and Goard, 2020; van Beest et al., 2021) and whisker-barrel (Nakai et al., 2017; Gilad et al., 2018; Gilad and Helmchen, 2020; Esmaeili et al., 2021), and motor learning (Makino et al., 2017; Kondo and Matsuzaki, 2021), decision making (Allen et al., 2017; Shimaoka et al., 2019; Salkoff et al., 2020) and multisensory integrations (Vanni and Murphy, 2014; Murphy et al., 2016; Vanni et al., 2017; Kuroki et al., 2018; Zhu et al., 2018). Notably, this technique is beneficial for the initial evaluation of cortical function in disease models (Balbi et al., 2017; McGirr et al., 2017; Nakai et al., 2017; Balbi et al., 2019; Cramer et al., 2019; Monai et al., 2021). For example, the group of Timothy Murphy at the University of British Columbia has demonstrated this technique to calculate functional connectivity in the murine cerebral cortex in both healthy and pathological brain states. Moreover, they have consistently reported that the distinct functional connectivity between the sensory and motor cortex appears to exhibit a regular spatiotemporal pattern (Vanni and Murphy, 2014; Murphy et al., 2016; Silasi et al., 2016; Balbi et al., 2019). Besides, the group of Fritjof Helmchen at the University of Zurich has reported that using the whisker-based texture discrimination task paradigm, distinct signal flow pathways can sustain short-term memory or refine sensory learning in mice. Furthermore, disrupting these separate routes can impair the performance of the mice (Gilad et al., 2018; Gilad and Helmchen, 2020). Therefore, transcranial cortex-wide imaging is likely to be a powerful tool for uncovering new features of brain dynamics. We selected the primary references pertaining to the cortex-wide transcranial calcium imaging and its practical applications, as outlined in Table 1.

**TABLE 1:** The primary references of the cortex-wide transcranial Ca^2+^ imaging.

References	Year	Indicator	What activity related to the phenomenon was visualized in the cortex?
Vanni and Murphy (2014)	2014	GCaMP3	Cortical functional connectivity
Xie et al. (2016)	2016	iGluSnFR	Regional cortical connectivity in spontaneous brain activity
Monai et al. (2016)	2016	G-CaMP7	Transcranial direct current stimulation-induced cortical plasticity
Silasi et al. (2016)	2016	GCaMP6s	Functional connectivity maps generated from spontaneous cortical activity
Murphy et al. (2016)	2016	GCaMP6s GCaMP6f	Functional cortical maps on the basis of both spontaneous activity and brief sensory stimuli such as light flashes
Allen et al. (2017)	2017	GCaMP6f	Decision-making behavior
Makino et al. (2017)	2017	GCaMP6s	Learning a motor task over s
Nakai et al. (2017)	2017	G-CaMP7	Whisker-barrel dysfunction in ASD model mouse
Balbi et al. (2017)	2017	GCaMP3	Focal ischemic stroke induction in target brain regions using photothrombosis
Vanni et al. (2017)	2017	GCaMP6s	Cortical activity parcellation
McGirr et al., 2017)	2017	iGluSnFR	Alternation of mesoscale glutamatergic networks after chronic stress and in response to the rapid acting antidepressant
Kuroki et al. (2018)	2018	YC2.60	Multisensory integration
Zhu et al. (2018)	2018	GCaMP6f	Active whisking and no whisking
Gilad et al. (2018)	2018	GCaMP6f	Whisker-based texture discrimination task with delayed response
Li et al. (2019)	2018	GCaMP6s	Functional parcellation of isocortex
Balbi et al. (2019)	2019	GCaMP6	Mesoscopic functional connectivity in a brain-wide microinfarct model
Michelson et al. (2019)	2019	GCaMP6s	To compare genetically encoded calcium sensors under transgenic or viral vector expression strategies
Shimaoka et al. (2019)	2019	GCaMP6s	Go/No Go visual detection task
Clancy et al. (2019)	2019	GCaMP6s	Correlation between the spiking of neurons in primary visual and retrosplenial cortex to Ca^2+^ activity across dorsal cortex
O’Hashi et al. (2019)	2019	G-CaMP7	Spontaneous activity as the functional connectivity maps
Brier et al. (2019)	2019	GCaMP6f	Spontaneous cortical activity in awake, anesthetized, and naturally sleeping mice
Cramer et al. (2019)	2019	GCaMP6s	Changes in inter- and intrahemispheric connectivity at multiple time points up to 56 days post-stroke and correlated them with behavioral deficits
Pinto et al. (2019)	2019	GCaMP6f	Evidence accumulation and visually guided tasks
Gilad and Helmchen (2020)	2020	GCaMP6f	Learning whisker-based texture discrimination
Salkoff et al. (2020)	2020	GCaMP6s	Go/NoGo visual detection task
Barson et al. (2020)	2020	GCaMP6f	Long-range cortical networks
Sit and Goard (2020)	2020	GCaMP6s	Perception of visual motion
Kondo and Matsuzaki (2021)	2021	R-CaMP1.07	Rewards and motor learning
van Beest et al. (2021)	2021	GCaMP6f	Population receptive-field (pRF) mapping
Monai et al. (2021)	2021	G-CaMP7	Cortical spreading depolarization
Esmaeili et al. (2021)	2021	RCaMP	Transforming a brief whisker stimulus into delayed motor response

## 3. Transcranial cortex-wide Ca^2+^ imaging with G7NG817 transgenic mice

Multiple transgenic mice expressing various GECIs have been developed for transcranial imaging, such as GCaMP3 (Vanni and Murphy, 2014; Balbi et al., 2017), GCaMP6f (Murphy et al., 2016; Allen et al., 2017; Gilad et al., 2018; Zhu et al., 2018; Balbi et al., 2019; Brier et al., 2019; Pinto et al., 2019; Barson et al., 2020; Gilad and Helmchen, 2020; van Beest et al., 2021), GCaMP6s (Murphy et al., 2016; Silasi et al., 2016; Makino et al., 2017; McGirr et al., 2017; Vanni et al., 2017; Zhu et al., 2018; Clancy et al., 2019; Cramer et al., 2019; Li et al., 2019; Michelson et al., 2019; Shimaoka et al., 2019; Salkoff et al., 2020; Sit and Goard, 2020), RCaMP (Esmaeili et al., 2021; Kondo and Matsuzaki, 2021), YC2.60 (Kuroki et al., 2018), and unique proteins such as the extracellular glutamate sensor iGluSnFR (Xie et al., 2016; McGirr et al., 2017).

**FIGURE 1:**
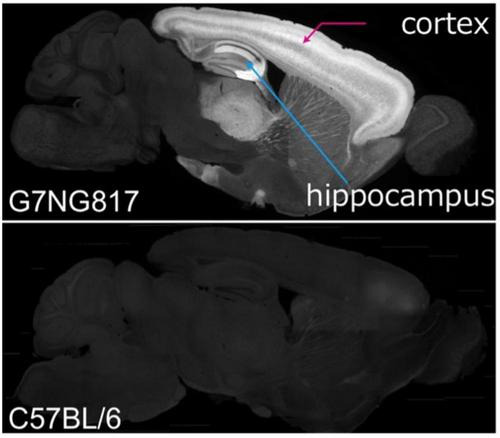
Fluorescent expression pattern of the sagittal section of fixed mouse brains. **(Top)** G7NG817 line transgenic mouse, **(bottom)** C57BL/6 line wild type mouse as a control [modified from Monai et al. (2016)].

In 2016, the group of Hajime Hirase at RIKEN generated a transgenic mouse line expressing G-CaMP7 (Ohkura et al., 2012) (the hyphen is officially needed), that is, G7NG817 mice (Monai et al., 2016), as shown in Figure 1. It has demonstrated the usefulness of G7NG817 mice in fully intact transcranial imaging (Monai et al., 2016; Nakai et al., 2017; Mishima et al., 2019; O’Hashi et al., 2019; Monai et al., 2021). The G7NG817 mouse expresses G-CaMP7 under the control of the glutamate transporter-1 (GLT-1) promoter to monitor the activity of cerebral astrocytes (Monai et al., 2016). GLT-1, known to be predominantly expressed on the astrocytic membrane for the uptake of glutamate, has been confirmed to be expressed in almost 100% of astrocytes and a subpopulation of excitatory neurons in the cortex. This expression pattern accurately reflects previously reported results (de Vivo et al., 2010), characterized by higher expression in layers 4 and 6, and then layer 2/3 (Figure 1). Additionally, we have confirmed that GABAergic interneurons and other types of glia, such as IBA-1 positive microglia, do not express G-CaMP7. Interestingly, the cerebral cortex expresses G-CaMP7 so densely that it enables observing cortical activity through the skull using standard epifluorescence microscopy.

**FIGURE 2:**
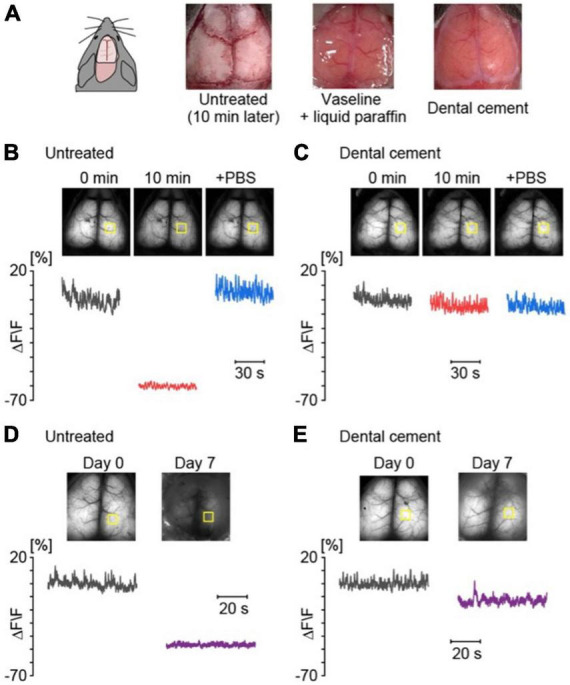
The skull treatment is necessary to obtain high-quality data for a long-term observation. **(A)** Examples of the skull surface without treatment and with Vaseline + liquid paraffine or dental cement. **(B)** Examples of cortical spontaneous activities for 1 min in an anesthetized G7NG817 mouse without treatment; just after removal of connective tissue (black), 10 min later (red), and after application of PBS to the dried skull surface (blue). The yellow squares indicate the region of interest (ROI) of the cortical region for the calculation of the fluorescence change ratios (ΔF/F). Each F value was calculated from the mean of 0 min. **(C)** Examples of cortical activities with application of dental cement on the skull surface, 0 min (black), 10 min (red), + phosphate-buffered saline (PBS) (blue), respectively. **(D)** Examples of cortical spontaneous activities for 1 min without treatment on day 0 (black) and day 7 (purple). **(E)** Examples of cortical spontaneous activities for 1 min with dental cement on day 0 (black) and day 7 (purple).

Given that the skull of mice is thin and transparent enough, the surface of the brain is visible through the cranial bone after opening the scalp. However, the transparency of the cranial bone is lost immediately after the removal of connective tissue from the skull (Figures 2A, B). To prevent drying, a mixture of liquid paraffin and Vaseline (1:1) was applied immediately after the removal of connective tissue from the skull surface. An alternative method is to treat the skull with dental cement, which helps preserve transparency for fluorescent imaging (Figure 2C). Without treatment, exposure of the skull for a week severely diminishes the Ca^2+^ signals for chronic imaging (Figure 2D). Thus, it is recommended to apply dental cement on the skull as soon as possible after the removal of connective tissue (Figure 2E). The materials, methods, and procedures are detailed in the Appendix.

**FIGURE 3:**
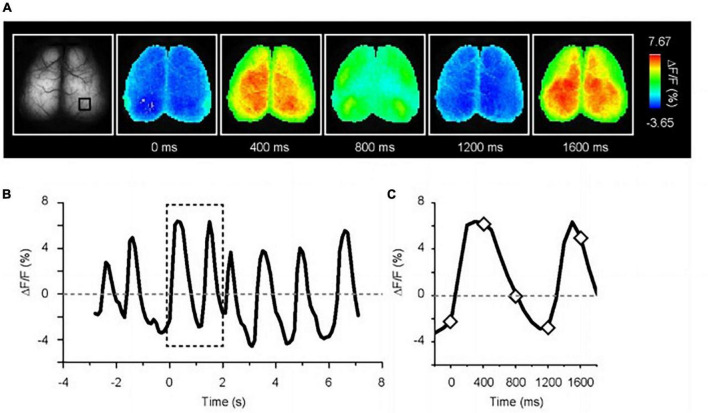
Spontaneous Ca^2+^ oscillation in deeply anesthetized G7NG817 mouse. **(A)** Representative images of Ca^2+^ oscillations. Pseudocolor is superimposed on the raw brain images. **(B)** Example trace of fluorescence change ratio in the black square drawn in **(A)**. The baseline is the mean of all-time course. The sampling rate is 10 Hz. **(C)** Magnified trace in the dotted line in **(B)**. The diamonds correspond to the time point shown in **(A)** [Modified from Monai et al. (2016)].

Figure 3 illustrates a representative example of spontaneous calcium oscillations in anesthetized G7NG817 mice, highlighting the usefulness of this mouse model for comprehensive imaging of cortical regions. The frequency of the up-down state was observed to be in the range of 0.5–0.2 Hz, which corresponds to the EEG known as a delta wave. Correspondingly, the areas between the left and right hemispheres were found to exhibit almost synchronous activity. Notably, active and inactive regions appeared to emerge alternately [see Monai et al. (2016), Supplementary Movie 1]^1^. Compared to the cortical map generated using voltage-sensitive dye imaging (Mohajerani et al., 2013), spontaneous activities under deep anesthesia in various sensory regions, such as visual or auditory, were observed randomly. Concurrently, it has been reported that general anesthesia significantly attenuates spontaneous astrocytic Ca^2+^ signaling (Thrane et al., 2012). Furthermore, as the kinetics of astrocytic Ca^2+^ dynamics are substantially slower than that of neurons, astrocytic Ca^2+^ contributes little to the spontaneous transcranial Ca^2+^ oscillations. Indeed, through two-photon microscopy, we observed cortical cellular activity during the spontaneous state and discovered that astrocytes exhibited almost no Ca^2+^ elevation under anesthesia.

## 4. Transcranial cortex-wide Ca^2+^ imaging during sensory stimulations

Transcranial imaging techniques allow for identifying regions within the brain that exhibit a response during specific sensory stimulation, a process referred to as cortical functional mapping. Researchers have employed various techniques to construct this map with voltage-sensitive dyes (Ferezou et al., 2007; Lim et al., 2012; Mohajerani et al., 2013) and Ca^2+^ sensors in sensory (Lim et al., 2012; Vanni and Murphy, 2014) and motor areas (Ayling et al., 2009; Hira et al., 2009).

**FIGURE 4:**
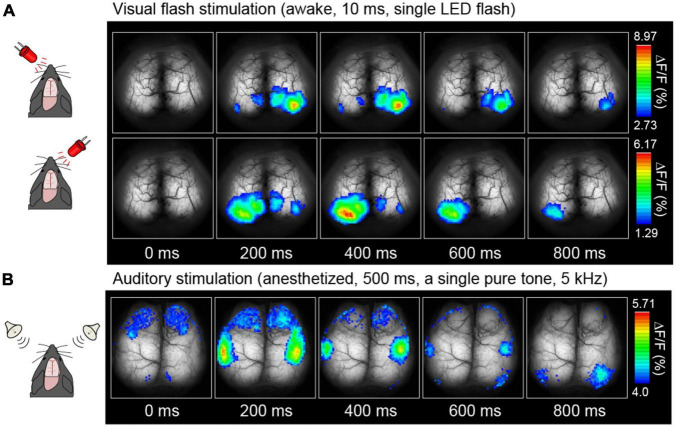
Examples of cortical sensory mapping. Visual stimuli **(A)** and auditory stimuli **(B)** were applied. Pseudocolor is superimposed on the raw brain images, which represents the fluorescence change ratio. The sampling rate was 30 Hz [Modified from Monai et al. (2016)].

**FIGURE 5:**
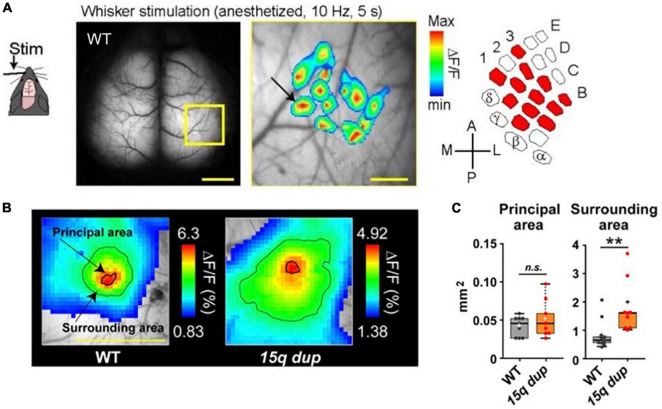
Examples of cortical whisker-barrel mapping in the WT mouse and autism spectrum disorder (ASD) mouse (15q dup). **(A)** A single whisker was vibrated for 5 s at 10 Hz. Pseudocolor is the mean of 16 trials and superimposed 12 whisker-barrel maps on one raw image, which is magnified in the yellow square. The drawn map is similar to the classically identified whisker-barrel relationship (right). Scale bar; 1 mm (left), 250 μm (center). **(B)** The different whisker-evoked area distribution in WT and 15q dup mouse. The bold line around the red area represents the principal area and a thin line on the green area represents the surrounding area. **(C)** Comparison of areas between WT and 15q dp. Data are shown in mean ± s. e. m. **p < 0. 01. [Modified from Monai et al. (2016), Nakai et al. (2017)].

Figures 4A, B, 5A depict examples of cortical functional mappings for various sensory modalities, including LED flash stimulation to the eyes (Figure 4A), pure tone stimulation to the ears (Figure 4B), and Piezo vibration stimulation to the whiskers (Figure 5A). These responses have been verified to be primarily of neuronal origin rather than astrocytic. The visual stimulus was administered via a single LED flash with a duration of 10 ms to each eye of unanesthetized mice, resulting in a rapid response within the primary visual cortices, 200–400 ms post-stimulation, on the left hemisphere for right eye stimulation and the right hemisphere for left eye stimulation, with a maximum amplitude of 10% (ΔF/F). No significant differences were observed in response time or amplitude in anesthetized mice. The audible range for mice is approximately 1–50 kHz. Thus a 5 kHz pure tone was employed as auditory stimulation for 500 ms in anesthetized mice, resulting in successful activation of the auditory areas on both hemispheres within sub-seconds (Figure 4B).

Additionally, a single whisker vibration at 10 Hz for 5 s was applied every 60 s to anesthetized mice using a syringe controlled by a piezoelectric device. The mean of 16 trials was calculated. Figure 5A presents a graphical representation of the superimposed 12-whisker responses on the corresponding barrel cortex, which accurately reproduces the known whisker-barrel relationship (Bernardo et al., 1990; Margolis et al., 2012).

## 5. Transcranial cortex-wide Ca^2+^ imaging with a disease model

These data were obtained from homozygous G7NG817 mice, although signals in heterozygous G7NG817 mice are sufficient for transcranial imaging. Consequently, generating multiple transgenics, such as disease models, is feasible through simple crossing with G7NG817, which will aid in the initial evaluation of any phenotype related to cortical functions.

For example, Nakai and Takumi generated a double transgenic mouse line comprising G7NG817 mice and one of the models for autism spectrum disorders (ASD), specifically the 15q dup mice (Nakatani et al., 2009), characterized by a duplication of the human 15q11-13 chromosomal region (Nakai et al., 2017). Previous studies have demonstrated that the 15q dup mice exhibit deficits in social communication and flexible behavior (Nakatani et al., 2009), and abnormal cortical spines and cerebellar functions (Isshiki et al., 2014; Piochon et al., 2014). In the ASD model, the evoked area in response to single whisker stimulation did not exhibit any notable differences in the “principal area,” which was defined as the region exceeding the mean response in a 3 × 3 binned area surrounding the peak value within 500 ms of stimulus onset. However, the “surrounding area” defined as the region displaying an amplitude more significant than 60% of the peak, showed a considerable enlargement and encroachment upon adjacent territories (Figures 5B, C). These findings suggest that the ASD model mice are impaired in accurately and correctly processing whisker sensory information in the barrel cortex (Figures 5B, C).

**FIGURE 6:**
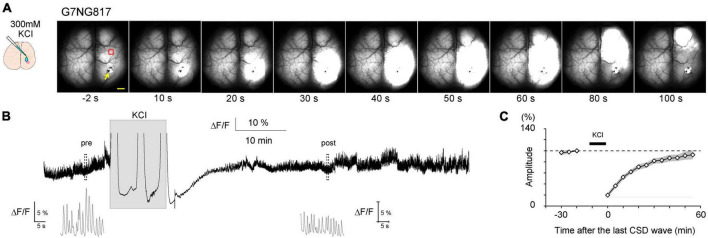
Typical example of a cortical spreading depolarization (CSD)-associated Ca^2+^ wave in an anesthetized G7NG817 mouse observed by transcranial cortex-wide macro imaging. **(A)** KCl (300 mM) was topically applied to a small craniotomy above the visual cortex at time 0. The yellow arrowhead points to the KCl application site. Representative Ca^2+^ dynamics shown in **(B,C)** were recorded in an ROI∼2 mm anterior to the KCl application site (red square). Scale bar 1 mm. **(B)** Example trace of Ca^2+^ signal (G-CaMP7 ΔF/F) from the same animal shown in **(A)**. Insets are magnified plots of the respective pre- and post-periods marked by dotted rectangles showing UP/DOWN state slow oscillations that occur during urethane anesthesia. [Modified from Monai et al. (2021)].

Next, the application of 300 mM KCl solution via a small craniotomy (∼2 mm) on the right hemisphere of G7NG817 mice for 10 min resulted in the generation of multiple Ca^2+^ waves that traversed the entire hemisphere (Figure 6). Cortical spontaneous activity was significantly attenuated for approximately 1 h following the removal of KCl. This phenomenon is known as cortical spreading depolarization (CSD), characterized by the propagation of aberrant neuronal excitatory waves at a velocity of approximately 4 mm/min (Leão, 1944). CSD has been linked to neurological disorders, including ischemic stroke, traumatic brain injury, post-seizure effects, and migraine aura (Lauritzen et al., 2011). Transcranial, cortex-wide Ca^2+^ imaging can aid in a deeper understanding of the properties of Ca^2+^ waves associated with CSD, including initiation, propagation, and recovery. The recovery speed of neural activity has been linked to astrocytic IP_3_/Ca^2+^ signaling (Monai et al., 2021). A study has shown that it generally took about an hour for the amplitude of the whisker stimulation-evoked LFP responses in the barrel cortex to fully recover in wild-type mice, but it took more than 3 h in a transgenic mouse lacking IP_3_ receptor type 2 (IP_3_R2KO mouse). However, the initiation and propagation speed was not affected in IP_3_R2KO mice (Monai et al., 2021).

## 6. Transcranial cortex-wide Ca^2+^ imaging of astrocytic activities and synaptic plasticity

**FIGURE 7:**
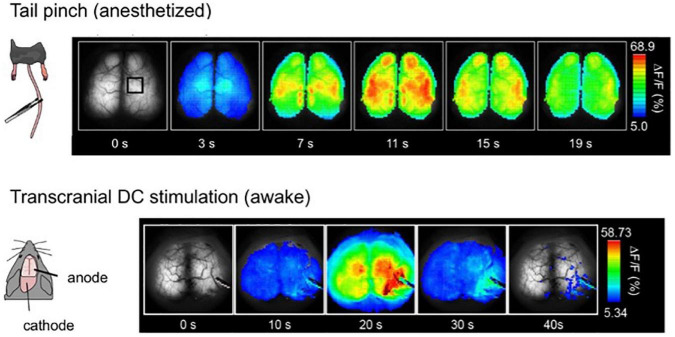
Global and slow response after tail-pinch and transcranial direct-current stimulation (tDCS). **(Top)** The tail of the urethane anesthetized mouse is pinched four times for 2 s at time zero. Pseudocoloring is superimposed on the raw images. **(Bottom)** Awake mice were fixed by their head with a metal head plate attached to the skull with dental cement. tDCS anode was placed on the right hemisphere, and the cathode was on the neck muscle. Weak direct current, 0. 1 mA, was applied for 10 min [Modified from Monai et al. (2016)].

A sharp tail pinch stimulation elicits a transition of EEG to a high-frequency wave, characterized by asynchronous, low amplitude, and approximately 40 Hz, referred to as the gamma rhythm. It was expected that tail pinch stimulation of urethane-anesthetized G7NG817 mice also resulted in a global transition to high-frequency and low-amplitude gamma rhythm of Ca^2+^ oscillation. However, in contrast to expectations, tail pinch stimulation resulted in a global response synchronized across the entire cortex, with an amplitude ten times higher and longer duration sustained for a few tens of seconds than other sensory stimulations. Furthermore, cellular-resolution observation using two-photon microscopy revealed that the bright and slow signals that appeared during tail pinch stimulation originated from astrocytes (Figure 7). The primary source of astrocytic Ca^2+^ elevation is the intracellular release from inositol 1,4,5-trisphosphate (IP_3_) receptors type 2 (IP_3_R2) located on the endoplasmic reticulum, triggered by IP_3_ produced through intracellular signaling induced by the activation of G-protein coupled receptors (GPCR) on the astrocytic membrane. Astrocytes possess a diverse array of GPCRs (Porter and McCarthy, 1997). The ligands of GPCRs are primarily neuromodulators, such as acetylcholine and noradrenaline. Tail pinch stimulation could elicit the release of multiple neuromodulators.

Next, applying a weak direct electric current stimulation to one hemisphere *via* the skull induced a similar Ca^2+^ response as tail pinch, slowly spreading across the entire cortex with high amplitude (Figure 7). This method is known as transcranial direct-current stimulation (tDCS). It has been reported that tDCS can alleviate various diseases, particularly psychiatric disorders such as depression [as reviewed in Monai and Hirase (2018)]. Furthermore, studies have demonstrated that tDCS can enhance learning and memory, with potential underlying neural mechanisms including facilitation of synaptic transmission, i.e., synaptic plasticity (Fritsch et al., 2010; Hasan et al., 2011; Hendy and Kidgell, 2013; Podda et al., 2016; Wu et al., 2017; Chan et al., 2021). Notably, the slow and bright Ca^2+^ response induced by tDCS is of astrocytic origin, and neurons do not exhibit a noticeable change during tDCS.

**FIGURE 8:**
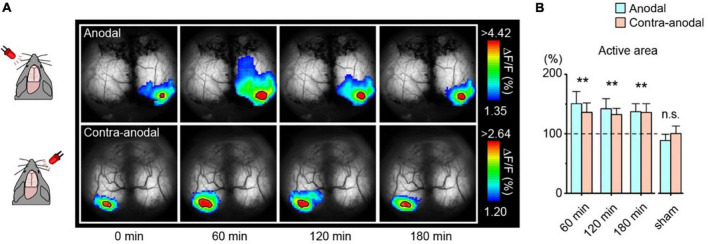
Enlargement of the visually evoked area after transcranial direct-current stimulation (tDCS). **(A)** Transcranial imaging of visual flash response after tDCS. The flash responses to left and right-eye are plotted as anodal and contra-anodal, respectively (ipsilateral responses are masked out). The color range is between mean + 1 SD and the peak value of the baseline visual evoked response. Solid black borders demarcate areas exceeding 90% of the baseline visual evoked response (active areas). **(B)** Comparison of the area of the visual-evoked region after tDCS. Data is mean + s. e. m. **p < 0. 01. [Modified from Monai et al. (2016)].

To visualize synaptic plasticity following tDCS, the LED-induced visual evoked area was repeatedly measured in awake mice (Figure 8A). The area significantly enlarged within 60 min after tDCS and persisted for at least 3 h (Figure 8B). Visual evoked potentials were also enhanced after tDCS as measured by electrophysiological techniques. It has been reported that astrocytic IP_3_/Ca^2+^ signaling plays an essential role in the mechanisms of tDCS-induced synaptic plasticity (Monai et al., 2016; Monai and Hirase, 2018; Mishima et al., 2019), suggesting that astrocytes play a far more significant role than previously thought in the brain, beyond mere support of neurons.

## 7. Conclusion

Transcranial cortex-wide imaging represents a robust methodology for the continuous, stable, and non-invasive monitoring of brain activity with a broad field of view. Notably, this technique is beneficial for the initial evaluation of cortical function in various transgenic mice, including disease models. Furthermore, as transcranial imaging does not require the opening of the skull, it enables the monitoring of long-term changes in neural activity, such as synaptic plasticity, in a less invasive manner. Presently, standard epifluorescence microscopy is utilized for transcranial imaging, although it has lower resolution and depth limitations than two-photon laser microscopy. It is hoped that the advancement of three-photon laser microscopy (Ouzounov et al., 2017; Wang et al., 2018) will permit observation of deeper regions at cellular resolution without requiring skull opening.

## Author contributions

SY and YW conducted the experiments, analyzed the data, designed the Figure 2, and contributed to the text. HM wrote the text and edited the all figures. All authors collaborated on the article and approved the final version.

## Footnotes

1
https://www.nature.com/articles/ncomms11100#Sec24
