# Supplementary material for: Transcranial cortex-wide Ca2+ imaging for the functional mapping of cortical dynamics
Source: Front Neurosci. 2023 Feb 15;17:1119793. doi: 10.3389/fnins.2023.1119793 (PMC9975744; doi:10.3389/fnins.2023.1119793)
Supplement: Supplementary file 1 [file Data_Sheet_1.PDF]

Appendix

Materials and Methods

All experimental protocols were approved by the Institutional Animal Care and Use Committee of Ochanomizu University, Japan (animal study protocols 22017). All animal experiments were performed according to the guidelines for animal experimentation of Ochanomizu University that conforms with the Fundamental Guidelines for Proper Conduct of Animal Experiment and Related Activities in Academic Research Institutions (Ministry of Education, Culture, Sports, Science and Technology, Japan). Efforts were taken to minimize the number of animals used. This study was carried out in compliance with the ARRIVE guidelines.

Surgical procedure

Adult male and female C57BL/6N mice were used (older than 8 weeks). Mice were housed under a 12 h /12 h light/dark cycle and raised in groups of up to five mice. Mice were anesthetized with urethane (1.6 g/kg) or ketamine/xylazine cocktail (70 mg/kg of ketamine and 10 mg/kg of xylazine), and the body temperature was maintained at 37 °C with a heating pad during surgery and recording.

In vivo transcranial imaging

Mice were fixed to a stereotaxic stage using an auxiliary ear bar to remove the effect of pulsation and respiration and placed under a fluorescence stereo microscope (MVX10, Evident). The U-MWB2 filter set (excitation 460-490 nm, emission 520 nm, Evident) was used with the U-HGLGPS light source (Evident). Images were acquired using the ORCA-Spark digital CMOS camera (Hamamatsu Photonics) using HC Image software (Hamamatsu Photonics). Images were acquired with a size of 512 × 512 pixels and 16 bit resolution and 10-Hz frame rate. For transcranial imaging, the skull was treated by a mixture of paraffin oil and Vaseline to increase transparency.
